# Supplementary material for: FKRP-dependent glycosylation of fibronectin regulates muscle pathology in muscular dystrophy
Source: Nat Commun. 2021 May 19;12:2951. doi: 10.1038/s41467-021-23217-6 (PMC8134429; doi:10.1038/s41467-021-23217-6)
Supplement: Supplementary file 1 — Description of Additional Supplementary Files [file 41467_2021_23217_MOESM1_ESM.pdf]

Title: Supplementary Movie 1:
Description: Confocal microscopy analysis of fibronectin and myosin10 co-localisation in the Golgi of control and patient myotubes. Video of Control, LGMD2I & CMD cells in order of appearance. Video is of myoblast trans-Golgi, cells stained: Golgi 58k marker (green), fibronectin (yellow) and myosin10 (cyan). Each layer is removed sequentially and video rotates around several viewing angles. The video reveals that in control cells fibronectin and myosin10 co-localise in the Golgi whereas this localisation is reduced in the disease muscle cell lines. This confocal data supports the 2D data acquired on STED. Furthermore, it is observed the myosin10 fails to correctly fragment in the disease cell lines when compared to control cells and reveals that there are larger connected volumes of myosin10 within the Golgi in the disease state.

Title: Supplementary Movie 2:
Description: 3D super resolution microscopy reveals fibronectin becomes trapped within the Golgi and does not interact with myosin-10 in patient cells. Video of Control, LGMD2I & CMD cells in order of appearance. Video is of myoblast trans-Golgi, cells stained: Golgi: Golgi Reassembly Stacking Protein 2 (Yellow), fibronectin (blue) and myosin10 in (green) captured using Airyscan super resolution microscopy. The Golgi is ghosted to display the interplay of fibronectin and myosin-10 within the Golgi. Fibronectin does not accumulate in the Golgi of the control cells but is miss-localised and retained within the Golgi of muscular dystrophy patient cell lines.
