# Supplementary material for: FKRP-dependent glycosylation of fibronectin regulates muscle pathology in muscular dystrophy
Source: Nat Commun. 2021 May 19;12:2951. doi: 10.1038/s41467-021-23217-6 (PMC8134429; doi:10.1038/s41467-021-23217-6)
Supplement: Supplementary file 2 — Supplementary Information [file 41467_2021_23217_MOESM2_ESM.pdf]

Supplementary Data and Methods.

Supplementary Figures and Legends.

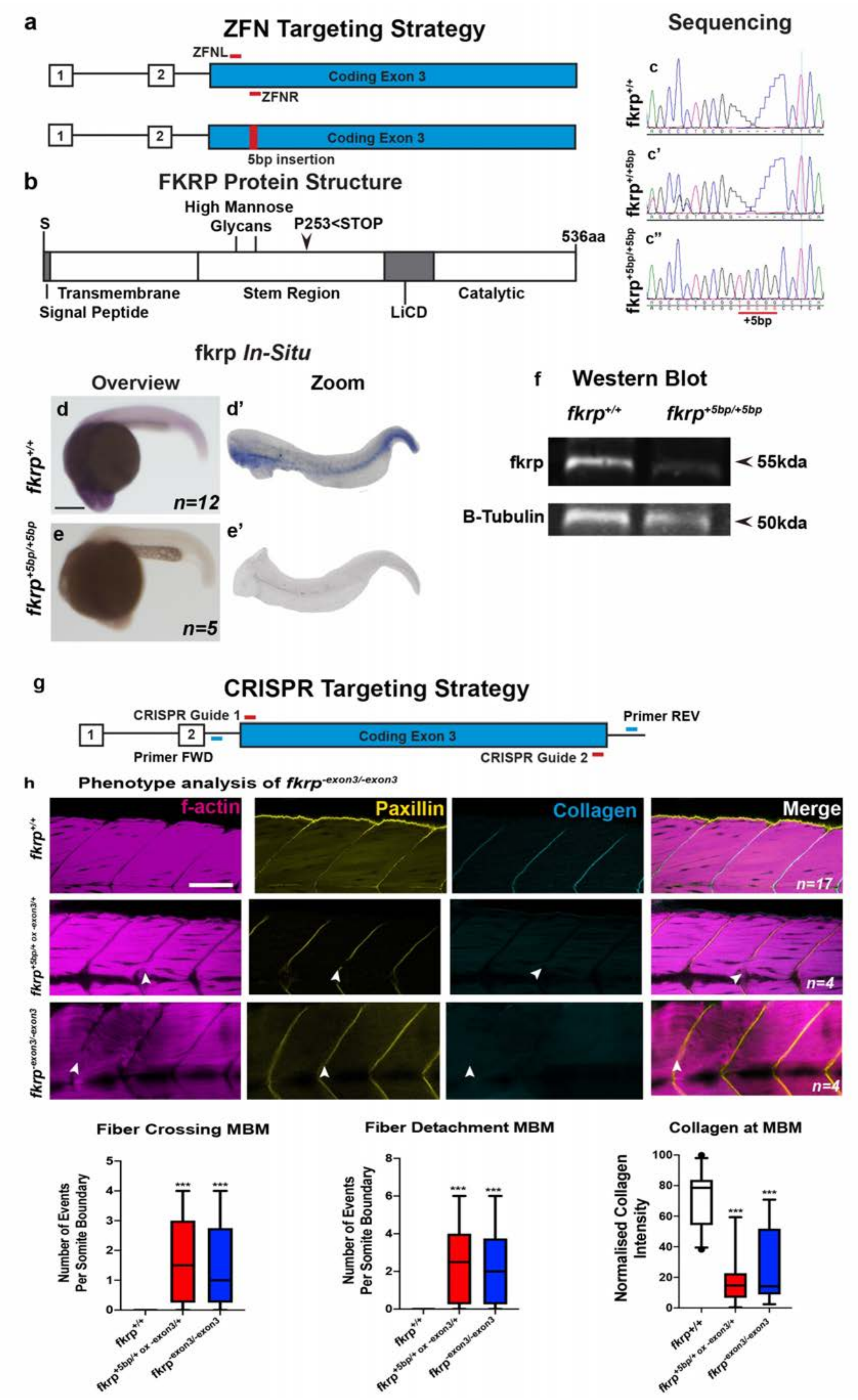

Supplementary Figure 1: Generation of a zebrafish *fkrp* loss of function model; *fkrp-/-* mutant alleles. **a**, Zinc Finger Nuclease **(**ZFN) targeting strategy to the start of the single coding exon (exon 3) of the zebrafish *fkrp* locus and resultant 5bp insertion. **b**, FKRP protein with position of premature stop codon at P253<STOP in the stem region of the protein, a region important for oligomerisation of glycosyltransferases, which would, if translated, produce a protein without an LiCD catalytic domain and thus would be predicted to contain no glycosyltransferase function. **c-c"**, Chromatograms of the 5bp insertion region aligned for **c**, *fkrp*$^{+/+}$ sibling, **c'**, *fkrp*$^{+/-}$ sibling and **c",** *fkrp*$^{-/-}$. **d-e**, 1dpf whole mount *in situ* hybridization (WISH) of *fkrp in* **d-d'**, *fkrp*$^{+/+}$ sibling and **e-e'**, *fkrp*$^{-/-}$ mutant embryos. The reduction of detected mRNA expression suggests that the zinc finger nuclease mutation results in the induction of a significant level of nonsense mediated decay. **f**, Western Blot analysis of lysate from 30 zebrafish per genotype, 30μg protein lysate in each lane, stained for antibodies against FKRP and Tubulin as a loading control, n=3. This analysis reveals the zinc finger induced allele, results in a loss of FKRP protein. **g**, Schematic of the second, *fkrp* CRISPR-induced, allele designed to remove the entire coding region of *fkrp* gene which is contained entirely in exon 3. Location of the crisper mRNA guides marked in red on the upper "blue exon" schematic. The location of the PCR genotyping flanking primers used in **h** to detect the removal of the entirety of exon three are marked in blue on the schematic. **h**, Phenotype analyses of heteroallelic cross between the two induced alleles results in a similar phenotype to either allele alone. The phenotype of the of the heteroallelic incross and the *fkrp-exon3/-exon3* crispr allele share the phenotype observed in the zfn fish line, namely fiber detachment and fiber crossing of the myoseptal boundary. Analyses included f-actin, collagen and paxillin stain at 5dpf, arrow points to fibre crossing myoseptal boundaries scale bar =100mm. **i-j**, Fibre crossing counts and fibre detachment per myosepta (MBM), respectively. **k**, collagen intensity normalised to maximum collagen in wild type controls, analysed in Fiji. **i-k**, box and whisker plot, middle line= mean, box= 95% confidence interval, error bars= SEM, using two-way ANOVA multiple

comparison assuming non-parametric data, significance of, ***(p<0.0001). The heteroallelic incross and the *fkrp-exon3/-exon3* crispr allele were both observed to have a significant drop ***(p<0.0001) in collagen at their myoseptal boundaries, phenocopying the *fkrp* zfn induced line described in the main text, three fish were examined from three repeats.

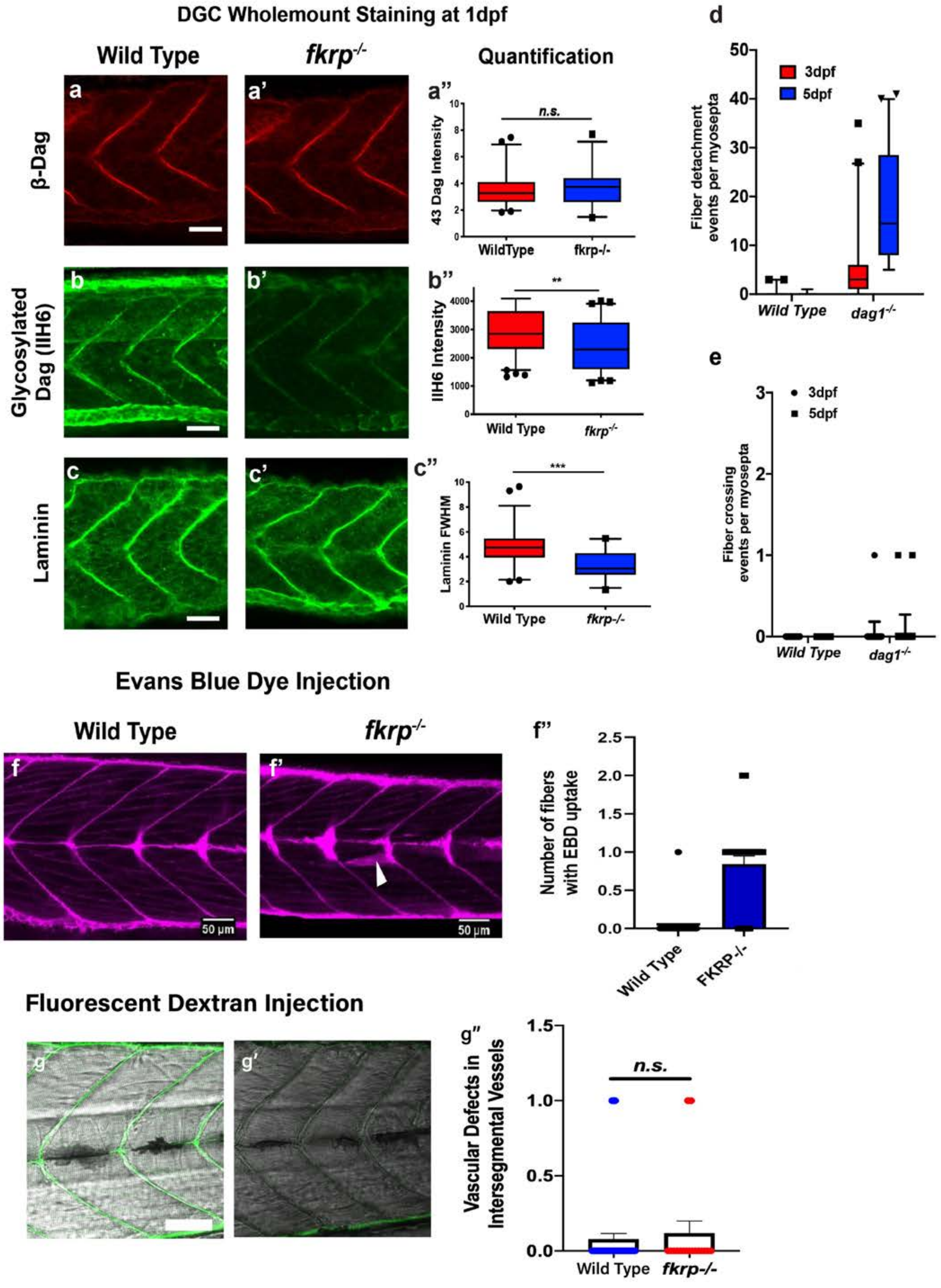

DGC Wholemount Staining at 1dpf
Wild Type
fkrp-/-
Quantification
β-Dag
Glycosylated Dag (IIH6)
Laminin
a
a'
a''
b
b'
b''
c
c'
c''
43 Dag Intensity
n.s.
WildType
fkrp-/-
IIH6 Intensity
**
Wild Type
fkrp-/-
Laminin FWHM
***
Wild Type
fkrp-/-
d
3dpf
5dpf
Fiber detachment events per myosepta
Wild Type
dag1-/-
e
3dpf
5dpf
Fiber crossing events per myosepta
Wild Type
dag1-/-
Evans Blue Dye Injection
Wild Type
fkrp-/-
f
f'
f''
50 µm
50 µm
Number of fibers with EBD uptake
Wild Type
FKRP-/-
Fluorescent Dextran Injection
g
g'
g''
Vascular Defects in Intersegmental Vessels
n.s.
Wild Type
fkrp-/-

Supplementary Figure 2: *fkrp*$^{-/-}$ mutant fish recapitulate patient DGC and fibre loss phenotypes. To evaluate the Dystrophin associated glycoprotein complex (DGC) in the *fkrp*$^{-/-}$ mutant fish, whole mount immunohistochemistry was carried out at 1dpf for core DGC components (a-c scale bar= 30µm). **a-a''**, Immunohistochemistry against β-dystroglycan (β-Dag) reveals no loss of dystroglycan protein in *fkrp* mutants (**a'**) compared to wildtype siblings (**a**). Quantitation in **a''** Wild Type (n=10) *fkrp-/-* (n=5). **b-b''**, Levels of glycosylated α-dystroglycan, which are dependent on FKRP activity were assessed using IIH6 antibody immunoreactivity, Wild Type (n=12) *fkrp-/-* (n=6). **c-c''**, laminin antibody was used since laminin is glycosylated dystroglycans core binding partner, Wild Type (n=15) *fkrp-/-* (n=9). **a-c**, **a'-c'**, Lateral wholemount staining and confocal imaging of zebrafish myotomes centred on anal pore and z-projected from 10 optical slices. **a''-c''**, Box plots with 95% confidence interval, points outside plotted individually, **a''-b''**, maximum intensity quantitation, ***(p<0.0001) **(p<0.001), **c''**, Full width half maximum analyses measured on Fiji image analysis software to quantitate the extent of laminin at vertical myosepta, box represents 5-95%, median center line, whiskers = SEM. **d**, Quantitation of fibre detachment events per myosepta analysed using two-way ANOVA multiple comparison assuming non-parametric data, box and whisker plot, middle line= mean, box= 95% confidence interval, **e**, Fibre crossing events per myosepta analysed using two-way ANOVA multiple comparison assuming non-parametric data. No significant difference was found by this analysis. **d-e**, error bars= SEM, points plotted outside 95% confidence interval, box represents 5-95%, median center line, whiskers = SEM. **f-f'**, Evans blue dye (EBD) injected 5dpf larvae, scale bar 50µm, reveals uptake globally in the vasculature and by a single muscle fiber (arrowhead, a marker of sarcolemma damage), but no other vascular leakage, wild type (n=12), *fkrp-/-* (n=5). **f'**, number of fibers per vertical myosepta, error bar= SEM 95% confidence interval, Student unpaired T-test analysis. **g-g''**, Angiography with fluorescent dextran injected into 5dpf larvae, scale bar 50µm, **g**, wild type (n=15), **g'** *fkrp-/-* (n=7). **g''**, number of breaks per vertical myosepta

intersegmental vessel, error bar= SEM 95% confidence interval, Student unpaired T-test analysis, scale bar = 30µm.

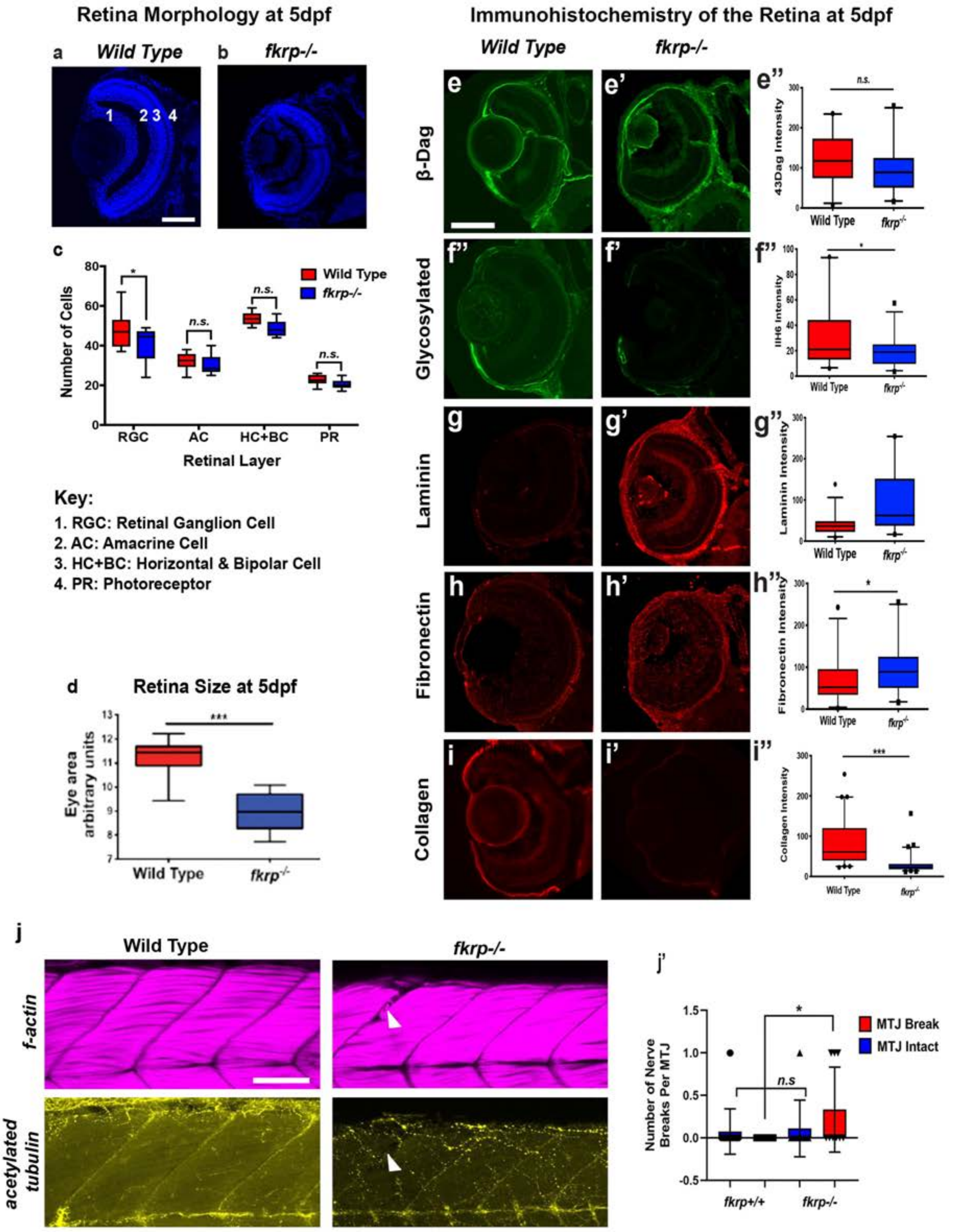

Retina Morphology at 5dpf
a Wild Type
b fkrp-/-
1 2 3 4
c
Number of Cells
Wild Type
fkrp-/-
n.s.
RGC
AC
HC+BC
PR
Retinal Layer
Key:
1. RGC: Retinal Ganglion Cell
2. AC: Amacrine Cell
3. HC+BC: Horizontal & Bipolar Cell
4. PR: Photoreceptor
d Retina Size at 5dpf
Eye area arbitrary units
Wild Type
fkrp-/-
Immunohistochemistry of the Retina at 5dpf
Wild Type
fkrp-/-
β-Dag
Glycosylated
Laminin
Fibronectin
Collagen
e e' e''
f'' f' f''
g g' g''
h h' h''
i i' i''
43Dag Intensity
IIH6 Intensity
Laminin Intensity
Fibronectin Intensity
Collagen Intensity
j Wild Type
fkrp-/-
f-actin
acetylated tubulin
j'
Number of Nerve Breaks Per MTJ
MTJ Break
MTJ Intact
n.s
fkrp+/+
fkrp-/-

Supplementary Figure 3: *fkrp*$^{-/-}$ mutant zebrafish recapitulate aspects of *FKRP* patient retinal phenotypes. **a-d**, Characterisation of retinal defects in *fkrp*$^{-/-}$ larvae using DAPI to mark retinal nuclei. **a, b**, Transverse cross sections through 5dpf retinas at the level of optic nerve stained with DAPI. **a**, Wild type sibling, **b**, *fkrp*$^{-/-}$. **c**, Quantification of the cellularity of different retinal layers in wild type sibling (red boxes) and *fkrp*$^{-/-}$ mutant retinas (blue boxes), *(P<0.05), (n=10) from three repeats, box represents 5-95%, median center line, whiskers = SEM. **d**, Quantitation of the overall size of *fkrp*$^{-/-}$ mutant and wild type retinas. Eye area was calculated in Fiji image analysis software by using the external most basement membrane as a marker of eye size, calculated on pixels arbitrary units ***(p<0.0001), scale bar=100µm. **e-i** Evaluation of Dystrophin associated glycoprotein complex (DGC) deposition at the myosepta including: glycosylated dystroglycan levels with IIH6 antibody, (n=6) from three replicates. **e,** Wholemount immunohistochemistry staining for β-dsytroglycan (β-Dag) on the retina. **f**, pan-Laminin **g**, fibronectin **h**, collagen1a **i**. *fkrp*$^{+/+}$ wild type sibling no dash, *fkrp*$^{-/-}$ single dash (') and double dash ("). **e-i**, Relative maximum intensity of staining at retina basement membranes: inner limiting membrane (closest to the lens) and Bruch's membrane otherwise known as the external limiting membrane (exterior encapsulating membrane), three measurements spaced equally apart in each membrane were taken in each fish at each membrane, scale bar=100µm. **e-i''** Box and whisker plot, middle line= mean, box= 95% confidence interval, error bars= SEM. **j-j',** Neural staining for acetylated tubulin (yellow) and f-actin staining (purple) in 5dpf larvae, arrow marks MTJ defect and neuronal breaks/absence associated with this defect, scale bar 50µm, *fkrp-/-* n=3, *wild type* (n=9). **j'** Analysis of neuronal deficits at myotendinous junction fiber breaks and in the myotome, student unpaired T-test *(p<0.05), error bars= 95% confidence interval SEM, scale bar = 50µm, (n=9) from three replicates.

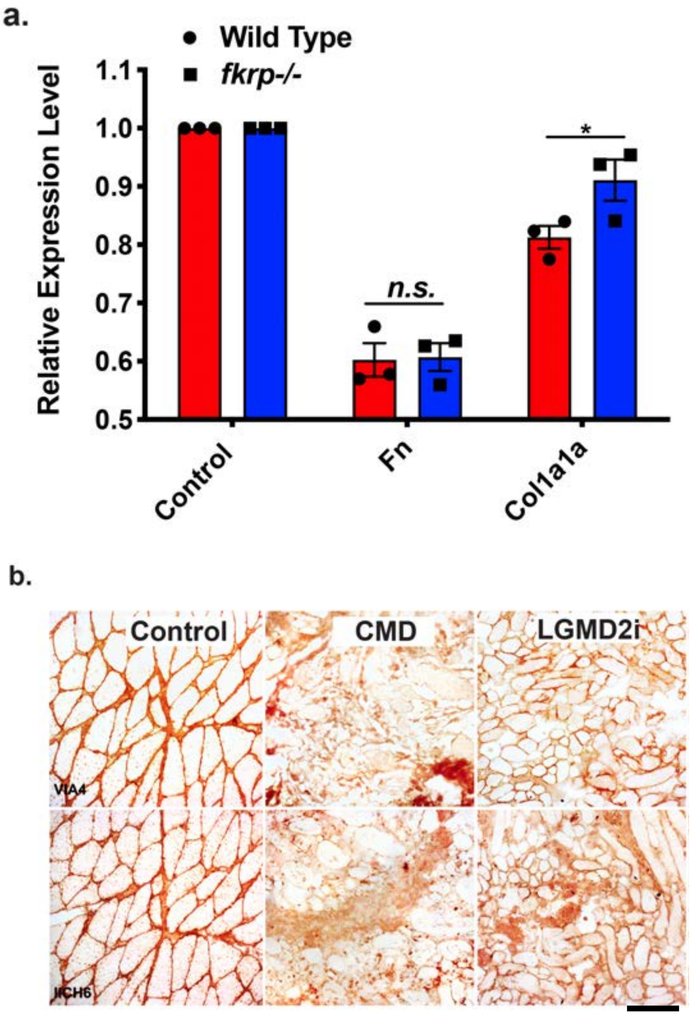

Supplementary Figure 4: *collagen* but not *fibronectin* gene expression is upregulated in *fkrp*$^{-/-}$ zebrafish. Glycosylation status of human cells on biopsy. a. Zebrafish *fibronectin* (*Fn*) and *collagen1a1a* (*Col1a1a*) gene expression levels from qPCR experiments, student t-test analysis between wild type and mutant, *(p=<0.05), student unpaired T-test analysis, (n=3) from three biological replicates and three technical replicates, average of technical data points shown, error bars = SEM. **b.** Patient biopsies of skeletal muscle, immunoreactivity of α-dystroglycan glycosylation with IIH6 and VIA4 antibodies. Scale bar=100µm, representative images from muscle biopsy at least three sections cut.

a: Fetuin *O*-glycans

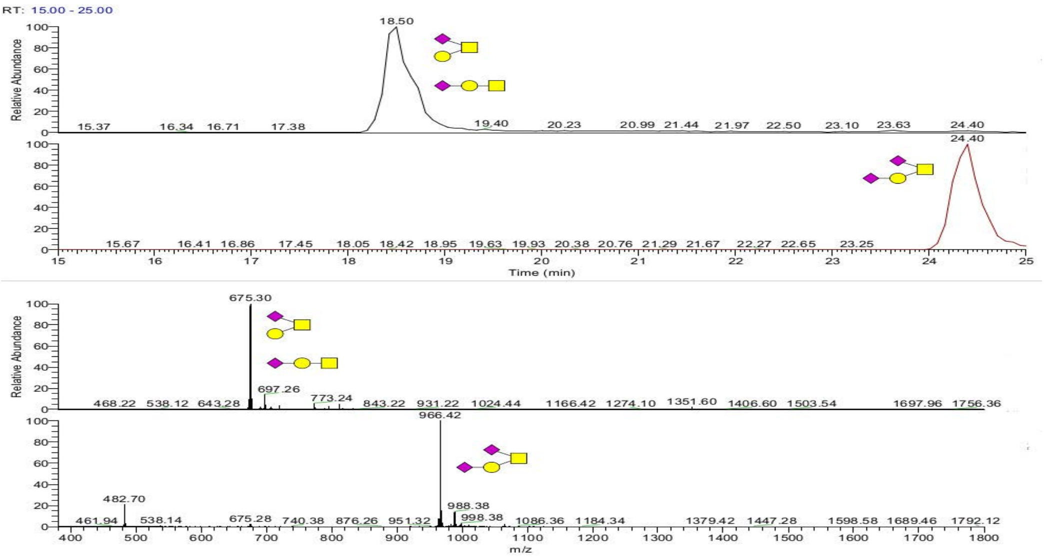

b: Fibronectin *O*-glycans

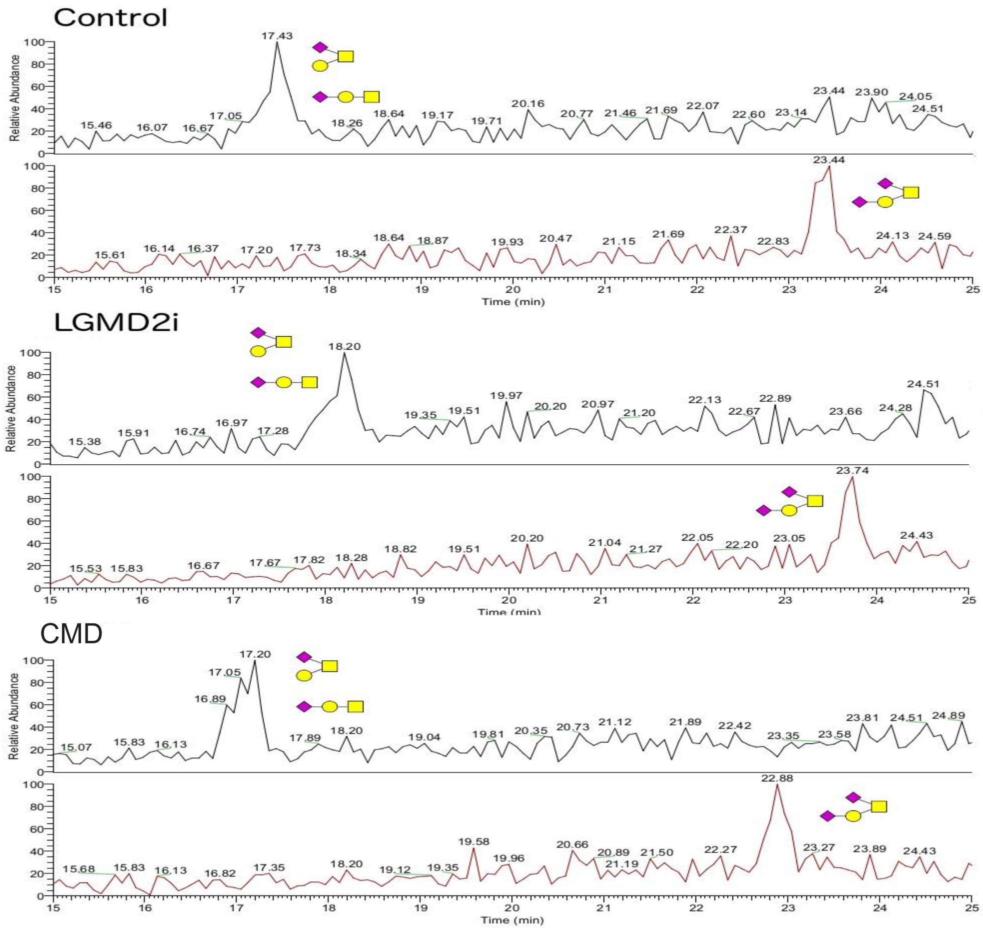

Supplementary Figure 5: Fibronectin *O*-Glycans are not altered in *fkrp* patient myotubes. LC *ms/ms* *O*-glycan composition of human cell fibronectin was determined in wild type control, LGMD2I, CMD human patient myotubes. **a**, Fetuin control analyses. Fetuin is a protein with a complete complement of *N* and *O* linked glycans that is commonly utilised for the standardisation of *ms/ms* glycan experiments[22]. This analysis demonstrates our technical ability to capture all relevant *O*-glycan signatures by LC *ms/ms*, **b**, fibronectin was immunoprecipitated from patient cells and *O*-glycans releases by the process of beta-elimination of serine linked mucin type glycans. Three human cell lines were examined; control patient sample, LGMD2I patient cells and cells derived from a congenital muscular dystrophy patient. No significant changes in *O*-glycan signatures could be observed via LC *ms/ms* analysis of fibronectins released from these human patient cell lines.

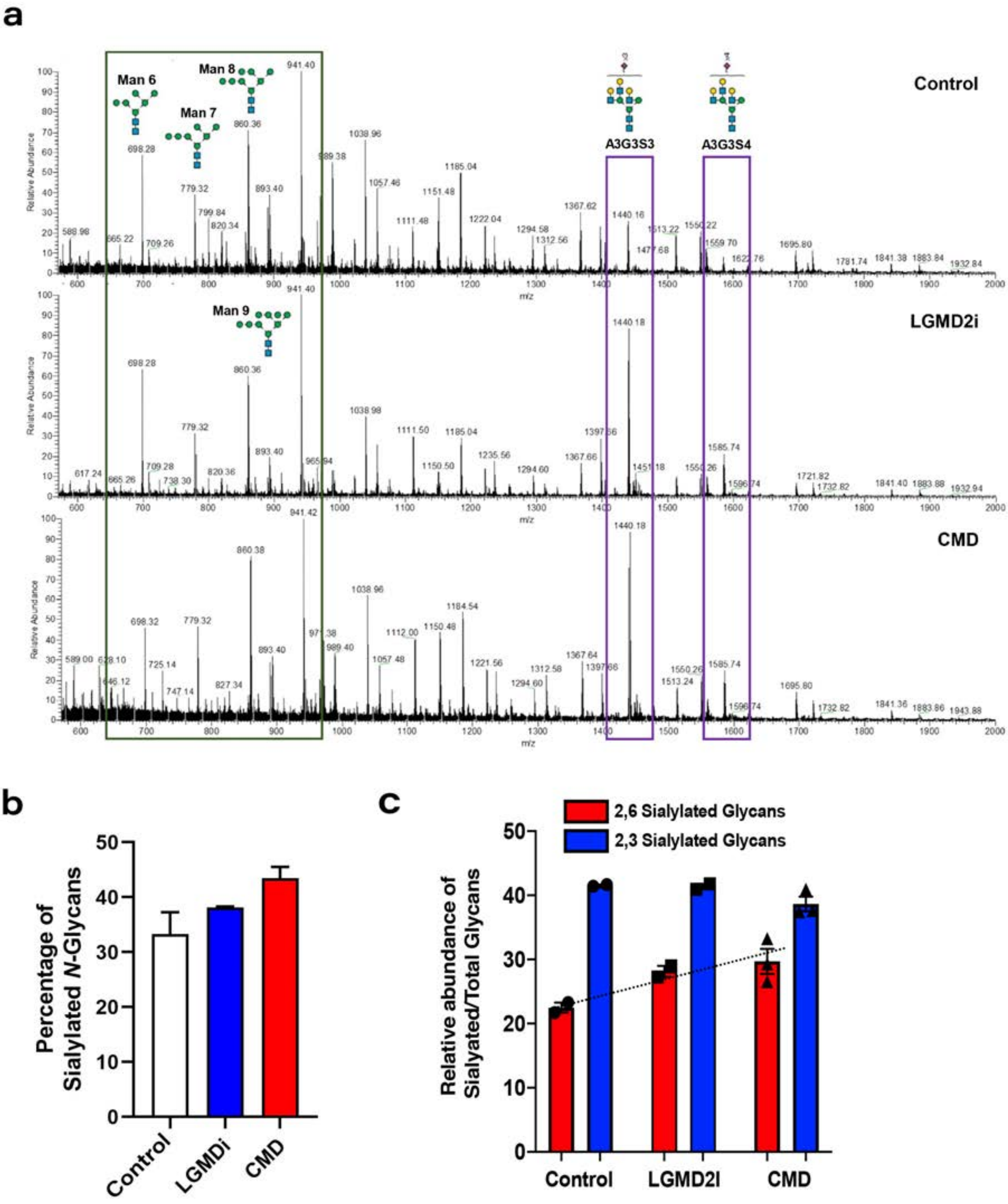

Supplementary Figure 6: *N*-Glycan analyses do not reveal a global deficit in sialylation in *FKRP* patient cells. To determine if the alteration to sialylation was specific for FKRP deficiency total cell lysates were examined for global sialylation levels. **a**, LC *ms/ms N*-glycan composition of healthy control, LGMD2I and CMD cells were determined. The *ms/ms* glycan analysis is presented graphically. Major peaks have been annotated with their predicted glycan structure. The results reveal that there is no discernible difference in peak heights or pattern

between different genotypes. **b**, Relative levels of sialylated glycans analysed via a one-way ANOVA, average of three technical replicates only, error bars = SEM. **c,** Analysis of 2,3 vs 2,6 sialylated glycans from the *ms/ms,* including a dotted trend line to indicate a general increase in 2,6 glycans in the patient samples[55], average of three technical replicates data points shown, error bars = SEM.

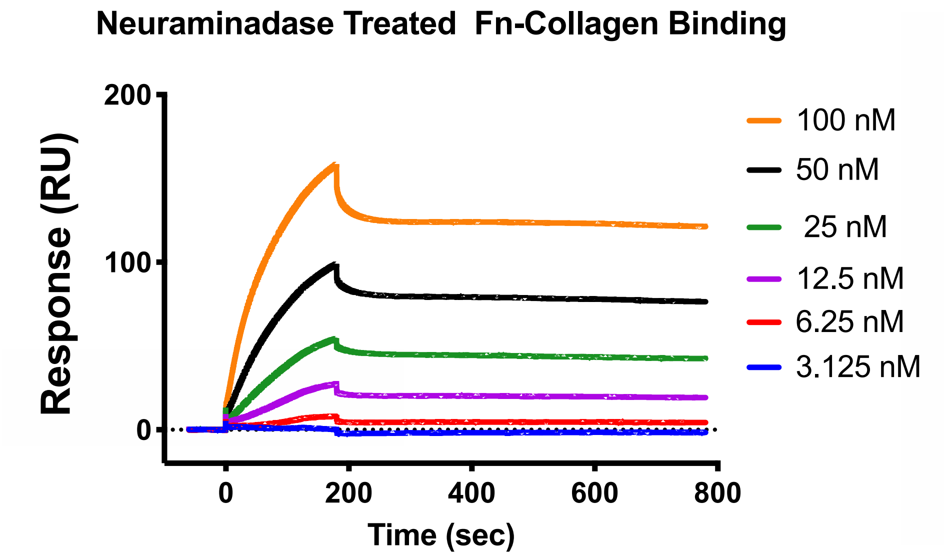

Supplementary Figure 7: Dose response of neuraminidase-treated fibronectin-collagen binding. Dose response curve for neuraminidase treated fibronectin (fibronectin concentrations 100nM-3.125nM) with the resulting collagen binding response assayed via Biacore analyses. Neuraminidase is an enzyme that removes sialic acid from the glycan structures. This analysis provides a control dose response analysis for Fig 3d, revealing that fibronectin binding status is not altered as collagen binding increases.

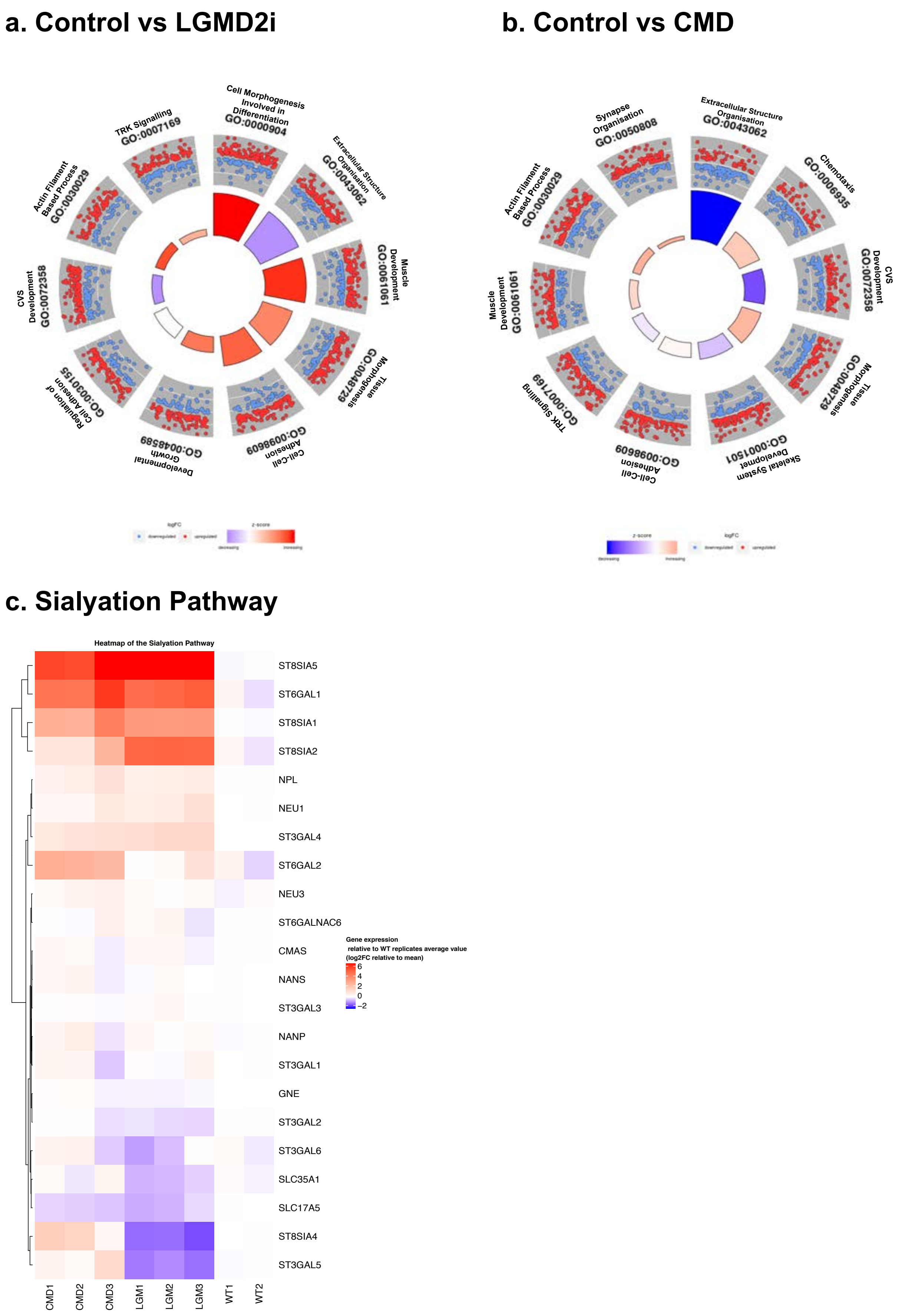
a. Control vs LGMD2i
Cell Morphogenesis Involved in Differentiation GO:0000904
Extracellular Structure Organisation GO:0043062
Muscle Development GO:0061061
Tissue Morphogenesis GO:0048729
Cell-Cell Adhesion GO:0098609
Developmental Growth GO:0048589
Regulation of Cell Adhesion GO:0030155
CVS Development GO:0072358
Actin Filament Based Process GO:0030029
TRK Signalling GO:0007169
b. Control vs CMD
Extracellular Structure Organisation GO:0043062
Chemotaxis GO:0006935
CVS Development GO:0072358
Tissue Morphogenesis GO:0048729
Skeletal System Developmet GO:0001501
Cell-Cell Adhesion GO:0098609
TRK Signalling GO:0007169
Muscle Development GO:0061061
Actin Filament Based Process GO:0030029
Synapse Organisation GO:0050808
c. Sialyation Pathway
Heatmap of the Sialyation Pathway
ST8SIA5
ST6GAL1
ST8SIA1
ST8SIA2
NPL
NEU1
ST3GAL4
ST6GAL2
NEU3
ST6GALNAC6
CMAS
NANS
ST3GAL3
NANP
ST3GAL1
GNE
ST3GAL2
ST3GAL6
SLC35A1
SLC17A5
ST8SIA4
ST3GAL5
CMD1
CMD2
CMD3
LGM1
LGM2
LGM3
WT1
WT2
Gene expression relative to WT replicates average value (log2FC relative to mean)
6
4
2
0
−2

Supplementary Figure 8: RNAseq analysis of LGMD2I and CMD patient cells. Top 20 enriched GO terms ordered by most changed pathway. **a**, Wild Type vs LGMD2I. **b**, Wild vs CMD. These analyses demonstrate that expected pathways are altered such as ECM organisation. **c,** sialyation pathway heatmap analysis. Heatmap analyses reveal that the sialylation genes responsible for addition of terminal sialic acid, such as *ST6GAL1,* and sialyltransferases that specifically sialylate galactose at ternary glycan structures, are significantly upregulated rather than down regulated as might be hypothesised based on loss of fibronectin sialylation in patient cells.

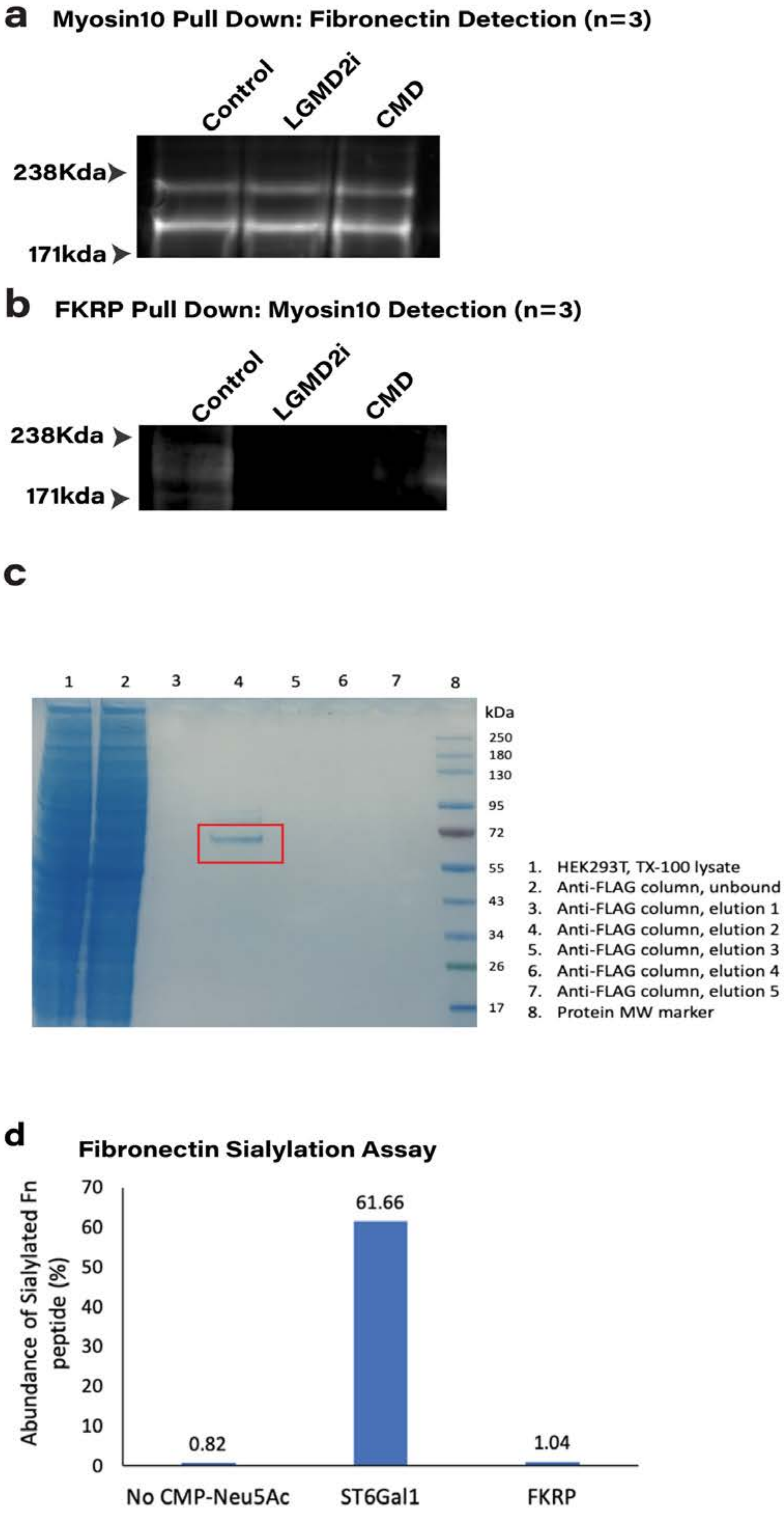
a Myosin10 Pull Down: Fibronectin Detection (n=3)
Control
LGMD2i
CMD
238Kda
171kda
b FKRP Pull Down: Myosin10 Detection (n=3)
Control
LGMD2i
CMD
238Kda
171kda
c
1 2 3 4 5 6 7 8
kDa
250
180
130
95
72
55
43
34
26
17
1. HEK293T, TX-100 lysate
2. Anti-FLAG column, unbound
3. Anti-FLAG column, elution 1
4. Anti-FLAG column, elution 2
5. Anti-FLAG column, elution 3
6. Anti-FLAG column, elution 4
7. Anti-FLAG column, elution 5
8. Protein MW marker
d Fibronectin Sialylation Assay
Abundance of Sialylated Fn peptide (%)
70
60
50
40
30
20
10
0
0.82
61.66
1.04
No CMP-Neu5Ac
ST6Gal1
FKRP

Supplementary Figure 9: FKRP binds myosin10 and has no detectable sialyation activity *in vitro*. **a,** Myosin10 co-immunoprecipitation of patient cell myotube membrane preparations, protein concentrations and volumes normalized prior to pull down. Fibronectin detected at approximately 220kda, two bands detected from the dimer in control, LGMD2I and CMD lysates (n=3). **b,** FKRP co-immunoprecipitation of patient cell myotube membrane preparations, concentrations and volumes normalized prior to pull down. Myosin10 detected at approximate 230kda in the control line and not detected in the LGMD2I and CMD membrane fractions (n=3)**. c,** Purification of human FKRP-FLAG protein from over expression in HEK293T cells**.** Coomassie blue stain with see plus 2 marker, bands appear between 55-72kda (red box), repeated 3 times, the shown image is the cleanest of the blots. **d**, Purified FKRP protein was incubated with desialylated fibronectin and cmp-sialic acid substrate to examine if FKRP possessed any sialylation activity relative to the known sialylating enzyme ST6Gal1. Sialyation assay, calculated from *ms/ms N*-glycan analysis of fibronectin via determining the area under *ms/ms* peak for sialylated vs non sialyated glycans released from fibronectin. This reveals that recombinant ST6Gal1 can add sialic acids to fibronectin in this assay but recombinant FKRP protein cannot.

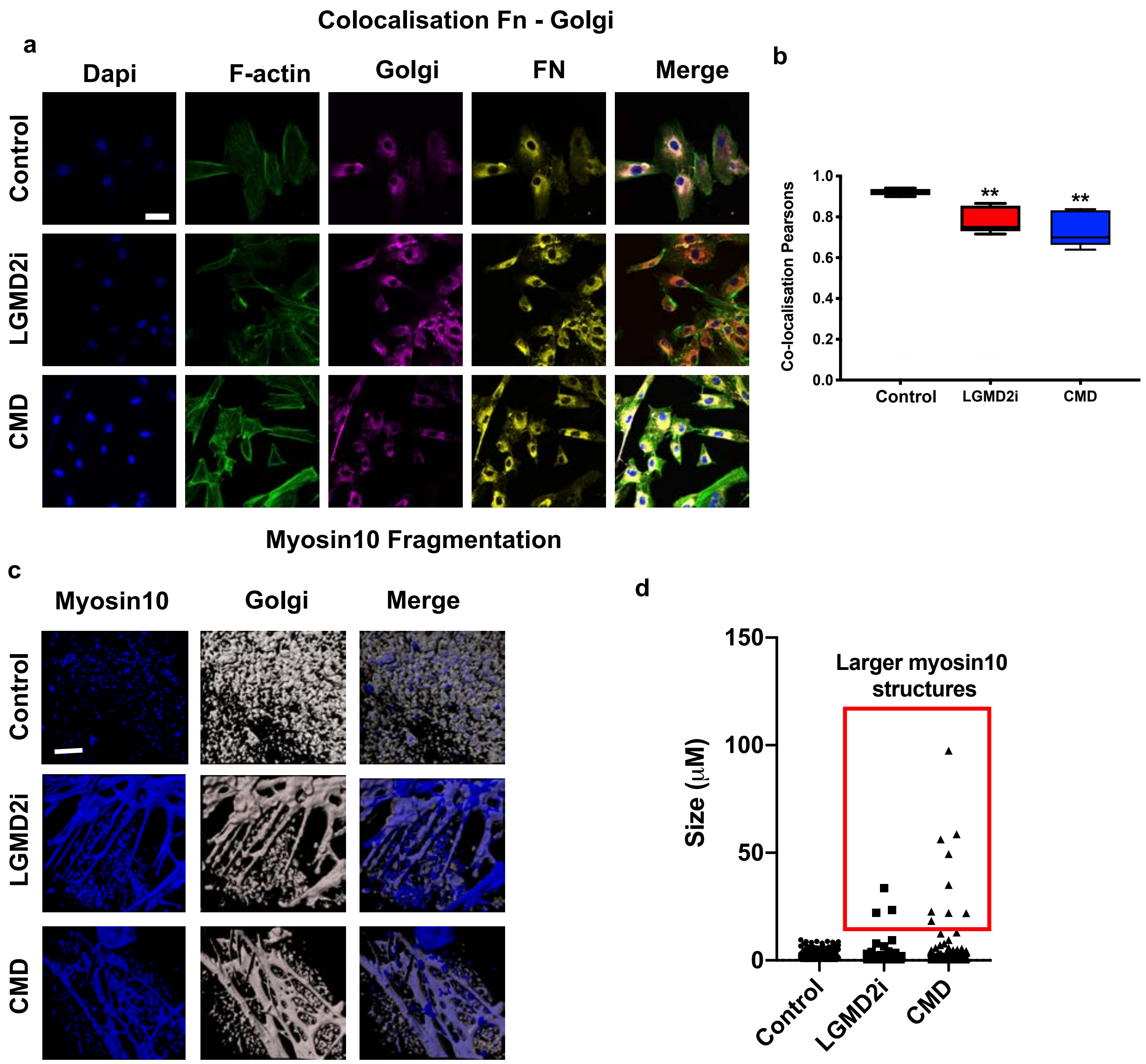

Supplementary Figure 10: Fibronectin is not correctly localized to the Golgi in patient cells. a, Confocal analysis of fibronectin (Fn) localisation to the Golgi, with f-actin and DAPI used as structural cellular markers (*n*=9), scale bar = 10µm. **b**, Pearson's co-localisation analysis of fibronectin to the Golgi, **(p<0.001) (*n*=9) analysed by one-way ANOVA, box represents 5-95%, median center line, whiskers = SEM. **c**, Airyscan of trans-Golgi 3D rendered in Imaris software, white 58k Golgi marker, blue myosin10, scale bar = 2µm. **d**, Rendered, volumes plotted to show fragmentation of myosin10 within the Golgi. Non-fragmented Myosin10 is regarded to be non-functional. Its fragmentation in trans-Golgi is the mechanism the Golgi uses to transport ECM components into the cytosol. The prevalence of larger

myosin10 structures (red box) specifically in patient myotubes indicates a defect in myosin10 compartmentalisation within these cells.

Supplementary Table 1.

Zebrafish	Forward	Reverse
**Col1a1a**	GAGGATGGTTGTACGTCGCA	AGGTGCACCAACGTCCATAG
**Col2a1a**	TCCTGCTACTTGTGGCAACG	GCTGGCCATCTTGAACACAG
**FN**	GTGCCACACACGTCTGAGTA	GTGCCACACACGTCTGAGTA
**zbact**	CGAGCTGTCTTCCCATCC	TCACCAACGTAGCTGTCTTTCTG
**zrp113a**	TCTGGAGGACTGTAAGAGGTATG	AGACGCACAATCTTGAGAGCAG
**ef1a**	GCTGGCAAGGTCACAAAGTC	GAACACGCCGCAACCTTTG
