# Supplementary material for: FKRP-dependent glycosylation of fibronectin regulates muscle pathology in muscular dystrophy
Source: Nat Commun. 2021 May 19;12:2951. doi: 10.1038/s41467-021-23217-6 (PMC8134429; doi:10.1038/s41467-021-23217-6)
Supplement: Supplementary file 5 — Reporting Summary [file 41467_2021_23217_MOESM5_ESM.pdf]

nature research

Corresponding author(s): Prof. Peter Currie

Last updated by author(s): Dec 10, 2020

Reporting Summary

Nature Research wishes to improve the reproducibility of the work that we publish. This form provides structure for consistency and transparency in reporting. For further information on Nature Research policies, see Authors & Referees and the Editorial Policy Checklist .

Statistics

For all statistical analyses, confirm that the following items are present in the figure legend, table legend, main text, or Methods section.

n/a	Confirmed	
☐	☒	The exact sample size (*n*) for each experimental group/condition, given as a discrete number and unit of measurement
☐	☒	A statement on whether measurements were taken from distinct samples or whether the same sample was measured repeatedly
☐	☒	The statistical test(s) used AND whether they are one- or two-sided<br>*Only common tests should be described solely by name; describe more complex techniques in the Methods section.*
☐	☒	A description of all covariates tested
☐	☒	A description of any assumptions or corrections, such as tests of normality and adjustment for multiple comparisons
☐	☒	A full description of the statistical parameters including central tendency (e.g. means) or other basic estimates (e.g. regression coefficient) AND variation (e.g. standard deviation) or associated estimates of uncertainty (e.g. confidence intervals)
☐	☒	For null hypothesis testing, the test statistic (e.g. *F*, *t*, *r*) with confidence intervals, effect sizes, degrees of freedom and *P* value noted<br>*Give P values as exact values whenever suitable.*
☒	☐	For Bayesian analysis, information on the choice of priors and Markov chain Monte Carlo settings
☒	☐	For hierarchical and complex designs, identification of the appropriate level for tests and full reporting of outcomes
☐	☒	Estimates of effect sizes (e.g. Cohen's *d*, Pearson's *r*), indicating how they were calculated

Our web collection on statistics for biologists contains articles on many of the points above.

Software and code

Policy information about availability of computer code

Data collection	All microscope systems, mass spectroscopy and biacore were controlled by the company specific proprietary acquisition software.
Data analysis	Image analysis- Fiji and Imaris 9.2 (Bitplane).<br>Statistical analysis- Prism version 7.0c (GraphPad Software, Inc.).<br>Metascape<br>GOplot (2, v1.0.2<br>Microsoft Excel V 14.4.6<br>Adobe photoshop 2020 V 21.1.2<br>Adobe illustrator CC 2018 V 22.1.0<br>Adobe premier pro 2020 V 14.1

For manuscripts utilizing custom algorithms or software that are central to the research but not yet described in published literature, software must be made available to editors/reviewers. We strongly encourage code deposition in a community repository (e.g. GitHub). See the Nature Research guidelines for submitting code & software for further information.

Data

Policy information about availability of data

All manuscripts must include a data availability statement. This statement should provide the following information, where applicable:

- Accession codes, unique identifiers, or web links for publicly available datasets
- A list of figures that have associated raw data
- A description of any restrictions on data availability

https://glycopost.glycosmos.org/preview/15008940036038edf6af4e9

(PIN CODE: 4292)
The GEO accession number for RNA-seq experiment is GSE163213 NCBI Gene Expression Omnibus

Field-specific reporting

Please select the one below that is the best fit for your research. If you are not sure, read the appropriate sections before making your selection.

- [x] Life sciences
- [ ] Behavioural & social sciences
- [ ] Ecological, evolutionary & environmental sciences

For a reference copy of the document with all sections, see nature.com/documents/nr-reporting-summary-flat.pdf

Life sciences study design

All studies must disclose on these points even when the disclosure is negative.

Sample size	No statistical methods were used to predetermine sample sizes. Sample sizes was chosen based on previous experience and standards in the field. Sufficient sample sizes were chosen for each experiment to determine whether the outcome was statistically significant.
Data exclusions	We excluded one replicate from our RNA seq analysis that failed stringency analysis
Replication	Experiments in fish were repeated in triplicate both technical and biological replicates as a minimum. Human samples were limited to technical replicates but ran in triplicates. We had one failed replicate on glycan mass spectroscopy analysis due to a technical error within the mass spec and excluded the result because it was not calibrated correctly.
Randomization	For general experiments and treatments of zebrafish larvae - larvae from several clutches were pooled and randomly allocated into groups.
Blinding	All fish experiments were run blind by analysis all samples before genotyping the fish. All biochemistry was blinded before passing to our colleagues in Sydney for ms/ms analysis. Imaging of cells was carried out blinded by our imaging facility.

Reporting for specific materials, systems and methods

We require information from authors about some types of materials, experimental systems and methods used in many studies. Here, indicate whether each material, system or method listed is relevant to your study. If you are not sure if a list item applies to your research, read the appropriate section before selecting a response.

Materials & experimental systems

n/a	Involved in the study	
[ ]	[x]	Antibodies
[ ]	[x]	Eukaryotic cell lines
[x]	[ ]	Palaeontology
[ ]	[x]	Animals and other organisms
[ ]	[x]	Human research participants
[x]	[ ]	Clinical data

Methods

n/a	Involved in the study	
[x]	[ ]	ChIP-seq
[x]	[ ]	Flow cytometry
[x]	[ ]	MRI-based neuroimaging

Antibodies

Antibodies used

Primaries:
fkrp: rabbit polyclonal 1:500 Invitrogen custom service (C + NPQYPNPAKKRLDRSR; 517-530)
1:500, Beta-Tubulin- Loading Control 50kda, IgG rabbit AbCam: ab6046, 1:10,000,
43DAG anti β-DG mouse IgG 1:50; Novocastra now Leica biosystems: PRODUCT ID: BETA-DYSTROGLYCAN,
IIH6 anti α-DG mouse IgM 1:50 Merc: 05-593)
pan-laminin anti rabbit IgG polyclonal 1:100; Sigma: L9393
Fibronection rabbit 1:200; Sigma F3648
Fibronectin mouse 1:250, abcam 2413
Collagen1 rabbit, Abcam, 1:100: ab34710
Golgi 58k Goat polyclonal ab23932.
Anti-non-muscle Myosin IIB/MYH10 antibody [3H2] ab684,GORASP
Secondaries:
Goat anti-mouse IgM Alexa Fluor 594: Invitrogen; A-21044, 1:500
Goat anti-mouse IgG Alexa Fluor 594, Invitrogen; A-21125, 1:500
Donkey anti-rabbit IgG Fluor 594, Invitrogen; 1:500
Alexa488 (Invitrogen: 1:250),
IRDye 800CW goat anti rabbit LI-COR: 926-32211, 1/5,000.

	goat anti mouse IgG Abberior580 1:250<br>Goat anti rabbit IgG Abberior635 1:250.
Validation	List of Validation:<br>Fkrp: Kawahara, G., Guyon, J. R., Nakamura, Y. & Kunkel, L. M. Zebrafish models for human FKRP muscular dystrophies. Hum Mol Genet 19, 623-633, doi:10.1093/hmg/ddp528 (2010).<br>Beta Tubulin: https://www.abcam.com/beta-tubulin-antibody-loading-control-ab6046-references.html#top-1033 600 references<br>IRD licor: https://www.licor.com/bio/reagents/irdye-800cw-infrared-dyes<br>43Dag: https://shop.leicabiosystems.com/us/ihc-ish/ihc-primary-antibodies/pid-beta-dystroglycan<br>IIH6: http://www.merckmillipore.com/AU/en/product/Anti-Dystroglycan-Antibody-clone-IIH6C4,MM_NF-05-593 Membrane organization of the dystrophin-glycoprotein complex.<br>Ervasti, J M and Campbell, K P<br>Cell, 66: 1121-31 (1991) 1991<br>Laminin: https://www.sigmaaldrich.com/catalog/product/sigma/l9393?lang=en&region=AUBasement membrane-like structures containing NTH α1(IV) are formed around the endothelial cell network in a novel in vitro angiogenesis model.<br>Yongchol Shin et. al<br>American journal of physiology. Cell physiology, 317(2), undefined (2019-6-13)<br>collagen1:RGD inhibition of itgb1 ameliorates laminin- α 2-deficient zebrafish fibre pathology<br>Alasdair J Wood, Naomi Cohen, Veronica Joshi, Mei Li, Adam Costin, Lucy Hersey, Emily A McKaige, Jessica D Manneken, Carmen Sonntag, Lee B Miles ... Show more<br>Human Molecular Genetics, Volume 28, Issue 9, 1 May 2019, Pages 1403–1413, https://doi.org/10.1093/hmg/ddy426<br>Myosin IIB/MYH10 antibody: Müller A et al. Actin stress fiber dynamics in laterally confined cells. Integr Biol (Camb) 11:175-185 (2019).<br>Golgi58k:Hofhuis J et al. Dysferlin mediates membrane tubulation and links T-tubule biogenesis to muscular dystrophy. J Cell Sci 130:841-852 (2017).<br>Secondaries were all well established antibodies.<br>All other primary antibodies were validated by the supplier for the purpose used in this manuscript.<br>If an antibody had not previously been tested in larval zebrafish tissue, its antibody expression pattern during developmental stages was compared to its in situ expression patterns to see if protein localisation corresponded to where its RNA transcripts were present.

Eukaryotic cell lines

Policy information about cell lines

Cell line source(s)	European Biobank: HEK293 were obtained from Sigma-Aldrich (Catalogue #:85120602)
Authentication	Lines were identified by genotype and immunohistochemistry analysis with IIH6 antibody
Mycoplasma contamination	All lines tested negative for Mycoplasma.
Commonly misidentified lines<br>(See ICLAC register)	No commonly miss identified lines were used in study. ILAC search performed.

Animals and other organisms

Policy information about studies involving animals; ARRIVE guidelines recommended for reporting animal research

Laboratory animals	Danio Rerio Zebrafish were used 1-7 days post fertilization, mixed gender. Adults fro breeding were kept upto 2 years of age strains used were TU wild type all transgenic and mutant lines were generated from TU strain.
Wild animals	Study did not involve wild animals.
Field-collected samples	Study did not involve samples collected from the field.
Ethics oversight	Monash Animal Ethics Comittee

Note that full information on the approval of the study protocol must also be provided in the manuscript.

Human research participants

Policy information about studies involving human research participants

Population characteristics	Infantile and adult patients affected with neuromuscular diseases, undergoing muscle biopsies for diagnostic purposes., at the Carlo Besta Neurological Institute, Milan, Italy.

Recruitment	Patients were recruited at the Carlo Besta Neurological Institute after informed patient / parental consent and in compliance with the Italian law. Samples were deposited, with informed consent, in the biobank "Cells, tissues and DNA from patients with neuromuscular diseases" for future studies.
Ethics oversight	Investigations on human tissue were approved by the Besta Institute review board and were in accordance with Italian law. Research on the cell lines obtained from the Carlo Besta Neurological Institute, at Monash University was conducted with ethical oversight and review via the Monash University Human Research Ethics Committee project umber CF14/3369 - 2014001793

Note that full information on the approval of the study protocol must also be provided in the manuscript.
